# Cultural Considerations in Suicide Risk Assessment with Immigrant and Refugee Populations in Canada

**DOI:** 10.1177/13634615251409709

**Published:** 2026-01-28

**Authors:** Moni Sadri-Gerrior, Kuppuswami Shivakumar, Nicolas Rouleau

**Affiliations:** 1 Department of Health Sciences, 8431 Wilfrid Laurier University, Waterloo, Canada; 2 Department of Psychiatry, 113744 Northern Ontario School of Medicine, Sudbury, Canada; 3 Department of Psychiatry, 60286 Health Sciences North, Sudbury, Canada

**Keywords:** suicide risk assessment, cultural awareness, immigrants, Canadian healthcare, self-harm

## Abstract

A suicide risk assessment (SRA) may predict and, in turn, prevent suicide; however, its efficacy is contingent upon assumptions that are built in to psychometrics and other tools with which risk is measured. In Canada, where multiculturalism is a celebrated part of national identity, specific populations including immigrants and refugees may be underserved by standard SRA protocols. Indeed, immigrant populations from minority cultures may express suicidality differently from non-immigrants in Canada and other Western countries. Here, we review the impact of culture on suicide and self-harm among immigrant populations within the historical and contemporary contexts of the Canadian healthcare system. We offer actionable recommendations such as the translation of SRA tools, standardization on relevant populations, and the inclusion of cultural factors in testing that can improve current SRA protocols, and discuss the implications of increased cultural awareness among clinicians.

## Introduction

Suicide is a leading cause of death globally and a major public health concern in Canada, where approximately 12 out of every 100,000 Canadians intentionally end their own lives each year (Statistics Canada, 2017a). As with many other medical conditions, not all suicides can be prevented; however, strategic actions by governments, public health organizations, and healthcare professionals have attempted to mitigate the causes of suicide and, in turn, its downstream effects on individuals, families, and communities. Unfortunately, media campaigns and increased public spending on mental health resources have had underwhelming effects on suicide prevention (Bridge et al., 2008; Rhodes et al., 2014; Saunders et al., 2017). Despite their modest influence, cost-effective efforts to raise public awareness of suicide, decrease social stigma related to mental illness, and encourage treatment-seeking behaviours are likely to receive continuing public support (Lebenbaum et al., 2020).

However, from the perspective of clinicians and healthcare providers, high-quality risk assessment is perhaps the most practical strategy to predict and mitigate self-harm and suicide. Suicide risk assessment (SRA) involves the identification of behaviours, thought patterns, previous trauma, chronic or acute conditions, and personal history associated with individuals who are suspected to be at risk of self-harm or suicide (Runeson et al., 2017). Importantly, protective factors including social support networks, coping mechanisms, and personal strengths are also part of a comprehensive SRA (Berman & Silverman, 2019). In 2011, the Ontario Hospital Association (OHA) and the Canadian Patient Safety Institute (CPSI) released detailed guidelines on SRA as a resource for healthcare organizations. In it, the OHA and CPSI highlighted cultural awareness as a foundational principle underlying SRA and integral to an accurate characterization of suicidality within the multicultural Canadian context. Suicide risk among immigrant and refugee populations in Canada is of particular concern, as most available SRA tools are inadequately designed to overcome barriers of language and culture, and the prevalence of suicide ideation in immigrant populations globally is a subject of concern. In a meta-analysis of 51 studies, Amiri (2022) found that 16% of newcomers have considered suicide, and as many as 6% have attempted it. While the Canadian Collaboration for Immigrant and Refugee Health (CCIRH) has provided general recommendations for newcomer preventative care (Kirmayer et al., 2011; Pottie et al., 2011), in the interests of providing quality mental health services to a diverse population, it is important that existing SRA strategies are adapted to address the needs of minority populations or that new tools are created to address gaps in care.

In recent decades, several reviews and meta-analyses have highlighted both the risks and protective features of culture for suicidality (Barker et al., 2017; Chu et al., 2010; Han et al., 2013; Kral, 2012; Yip et al., 2015). Indeed, there are considerably variable suicide rates across ethnic, gender, age, sexual orientation, and religious groups, among others (Chu et al., 2010). Clinicians are therefore faced with the challenge of striking a balance between personalized medicine and standard practices. Manifestations of suicidal behaviour and thought patterns are expressed differently across cultural groups; consequently, unique sets of questions and specialized metrics have been developed to capture and predict suicide and self-harm risk among specific groups (Chu et al., 2013). SRA needs to be coupled with an understanding of how different cultures view, express, and cope with suicide and its proximal causes—replacing a one-size-fits-all model of clinical assessment with focused strategies for minority populations.

This article examines the need for the development and application of culturally sensitive SRA tools that can be used with immigrant and refugee populations from minority cultural backgrounds within the Canadian healthcare system. Following a brief review of the impact of culture on suicide, we discuss the pressing need to provide SRA in languages other than English or French, establish norms based on data from specific populations, and develop brief culturally appropriate clinical tools with adequate sensitivity and specificity that can be used by healthcare workers, policymakers, and researchers.

## How Suicide is Affected by Culture: A Brief Review

Re-emphasizing the importance of Durkheim's (1897/1970) seminal research that demonstrated the predictive power of the organizational features of culture on decreased rates of suicide, contemporary researchers have further delineated the influence of culture on the behaviours associated with suicide as well as its proximal causes and correlates (Lester, 2009). Notably, Chu et al. (2010) have provided a cultural model of suicide in which they identified four factors that could account for 95% of culturally specific risk data in the North American literature. Of 240 empirical research findings identified as culturally specific, 228 could be categorized into the four factors (Chu et al., 2010).

The first factor, *cultural sanctions*, refers to how acceptable suicide is as an option within the culture as well as how an individual grapples with feelings of shame. The acceptability of suicide within one's religious tradition, in particular, has served as a strong predictor of suicidality (Anglin et al., 2005; Durkheim, 1897/1970; Stack & Kposowa, 2011). Whereas most cultures express a clear taboo against suicide, moral justifications and prescriptions can be found within a subset of cultural contexts such as in the cases of euthanasia, seppuku, and terrorist martyrdom (Pierre, 2015). From a clinical perspective, the patient's view of suicide, as informed by their cultural background, should be a strong determinant of approaches to SRA. Norm-referenced response patterns may not reflect the culturally specific attitudes of first-generation immigrants in particular.

The second factor, *idioms of stress*, involves the expression of suicidality, the visibility of warning signs, as well as the means or methods associated with suicide attempts. Indeed, it has been reported that minority groups can be less likely to self-disclose negative feelings; these individuals have been referred to as hidden ideators within the clinical context (Morrison & Downey, 2000). In his classic paper, Nichter (1981) drew attention to how high-caste women in rural India used specific phrasing and behaviours in response to distress otherwise unseen. Having coined these culturally specific behaviours as idioms of distress, we can similarly see how other cultures, ethnic groups, and communities express these idioms in unique ways (Kaiser & Weaver, 2019). Symptoms of depression, for example, can be expressed in either psychological or somatic terms for Western (i.e., Euro-Canadian) and Eastern (i.e., Chinese) cultures respectively (Ryder et al., 2008). Depending upon their cultural background, a patient may describe mood disturbances in terms that differ radically from the typical response patterns observed among members of the majority culture. Indeed, depression may be reported as “feeling blue,” and may incorporate concepts of shame or sinfulness or even the physical sensations of one's heart being squeezed (Jayawickreme et al., 2012; Keyes & Ryff, 2003). Similarly, the use of substances prior to or as a means of suicide can vary significantly across individuals from different cultural backgrounds (Chu et al., 2010). Fundamentally, it is important to look not only at the individual act of suicide but also at the broader cultural, political, and social contexts for each patient (Kirmayer, 2022). The assessment of risk is one of a clinician’s most important tasks and is chiefly informed by predictive factors. Misinterpreting behaviour or the results of classical psychometric inventories due to a lack of cultural awareness can spell disaster for patient care.

The third factor, *minority stress*, points to stresses born of discrimination, social disadvantage, acculturation or assimilation, and notions of social hierarchy. Importantly, the process of acculturation itself may be significant as it is typically those individuals who find themselves simultaneously distanced from both the majority culture and their parents’ culture of origin—often second-generation immigrants—who are found to be at higher risk of suicide (Berry & Sabatier, 2010; Di Thiene et al., 2015; Hjern & Allebeck, 2002; Law et al., 2014; Portes & Zhou, 1993). Substance use—which can begin as a means to cope with stress—among immigrant populations compounds suicide risk (Peña et al., 2008). Harassment, discrimination, internalization of negative stereotypes, and other forms of mistreatment are also strong predictors of suicide among minority cultures (Chu et al., 2010). An appreciation of the environment within which the patient is situated and whether it is likely to contribute to minority stress is highly relevant to SRA.

The fourth and final factor, *social discord*, refers to an individual's sense of belonging within their family, community, and friend circles. How suicide risk and types of social discord interact can vary significantly across cultures. While data specific to minority cultures in Canada are limited, in the American context family conflict is the type of discord most associated with individuals of Asian or Latino backgrounds (Cheng et al., 2010; Chung, 2003), while the integrity of informal social connections or relationships with extended family is uniquely predictive of suicide risk among individuals of African descent (Stack, 1996). Similar relationships are likely to exist among immigrant and refugee populations in Canada. SRA tools and strategies must be sensitive to culturally specific factors to accurately compute risk and form a basis for intervention.

## Minority Culture and Mental Health Issues in Canada

Clinicians must be able to simultaneously consider the proximal causes of crisis that patients experience and the historical truths that have contributed to systemic sources of stress. Without such considerations, it can be difficult to establish accurate attributions of major warning signs of suicide including hopelessness, revenge seeking, recklessness, substance abuse, and social withdrawal (Rudd et al., 2006). Despite enjoying a modern reputation for tolerance and inclusion, like many other countries, Canada has historically carried out or endorsed efforts to erode, eliminate, or replace minority cultures. Historical woes such as the racially motivated denial of entry of Indian immigrants aboard the Komagata Maru in 1914 and the subsequent death of 20 Sikhs upon their return (Johnston, 2014) contrast with Canada's modern status as a world leader in refugee resettlement (Basok & Simmons, 2016). Indeed, multiculturalism—and often the popular celebration of minority cultures—is an integral part of the modern Canadian identity (Ghosh, 2018). However, immigrants to Canada, including refugees, have reported deterioration of physical and mental health after settling in the country (De Maio, 2010; Mulé, 2020). Similarly, long-lasting negative impacts on mental health assessment (Smith & Kobayashi, 2002), as well as social structure and inclusion (Sakamoto et al., 2016), associated with past atrocities such as the internment of Japanese Canadians during the Second World War highlight the importance of cultural context in clinical practice. Fortunately, emerging research in Canada has increasingly sought to identify predictors of mental health status in immigrant populations (Browne et al., 2017; Emerson & Carbert, 2019).

## Current Status of Suicide Risk in Immigrant and Refugee Populations in Canada

Saunders et al. (2019) have provided recent data indicating that suicide rates among immigrant populations in Ontario, Canada are lower than those of long-term residents. One likely explanation for this encouraging observation is the Healthy Immigrant Effect, a positive selection phenomenon wherein recent immigrants are typically observed to be healthier than the non-immigrant population in the host country. The effect is driven by the fact that the immigration system typically selects those who are physically and socioeconomically able to migrate. Nevertheless, the same comprehensive population-based cohort study by Saunders et al. (2019) revealed that country of origin was significantly associated with suicide rate. While the suicide rates of immigrants from non-English-speaking countries such as Russia and Sri Lanka were comparable to those of non-immigrants, reduced risk was observed among immigrants from India and China (Saunders et al., 2019). In some instances, suicide risk for immigrants may also correlate with the length of time spent in their arrival country (Brenes, 2023). A systematic review conducted by Tyler et al. (2022) suggested that newcomers experienced migrant-specific stressors that may have increased their likelihood of displaying suicidal ideation. Refugees, notably, were more than twice as likely to die by suicide and nearly three times as likely to self-harm relative to non-refugee immigrants.

These data have implications for SRA. First, newcomers are not a monolith; therefore, the development of culturally sensitive SRA tools and strategies needs to consider the country of origin and refugee status. Second, despite the fact that suicide and self-harm risk is lower among immigrants as a group relative to long-term residents (in Ontario, Canada), from a clinical perspective it is still imperative that SRA tools be available to address the needs of individual patients. That is to say, individual immigrants from countries displaying low group averages of suicide risk must still be assessed clinically in the event of a suspected or anticipated crisis. It should also be noted that there is conflicting data surrounding suicide rates in first-generation versus second-generation newcomer populations. Studies in Sweden have shown that second-generation immigrants have higher rates of suicide compared to non-immigrants and first-generation immigrants who may experience the Healthy Immigrant Effect (Di Thiene et al., 2015; Hjern & Allebeck, 2002). However, in the Canadian context, there is inconclusive data regarding differences in immigrant generation and suicidality (Kennedy et al., 2005).

## Improving SRA for Immigrants in the Canadian Healthcare System

Existing recommendations for the incorporation of cultural influence into SRA have highlighted the importance of acknowledging minority cultures, including the appropriate standardization of SRA instruments and paying careful attention to cultural issues and predictors that might otherwise not be assumed (Westefeld et al., 2008). Building on these, we propose three actionable recommendations that will increase the quality of SRA for immigrants in the Canadian healthcare system.

### SRA Tools Must Be Translated to Languages Other Than English and French

The Beck Depression Inventory is one of a handful of gold-standard psychiatric assessment instruments that is commonly used for SRAs (Adler et al., 2015). While the test has been officially translated into several non-English languages (e.g., Arabic, Chinese, German, Italian, Turkish, Xhosa, etc.), it has not been officially translated to any of the major non-English languages of India (e.g., Hindi, Bengali, Telugu, Tamil, Gujarati, etc.). This is an unfortunate challenge for clinicians in Canada given India's position among the top 10 reported ethnic origins in Canada (Statistics Canada, 2017b). We recommend the translation (or if necessary, creation) of more SRA tools into a variety of languages across multiple cultures to better encompass the cross-cultural validity necessary in Canada's multicultural environment. Thus far, this has proven to be successful by the Columbia-Suicide Seversity Scale (C-SSRS), which has been translated into and validated across over 140 languages and populations (Oquendo & Bernanke, 2017). To best capture the subtleties that each language presents as a unique means of measuring psychometric indicators, we recommend that translations should be completed in partnership with members of the community who both speak the language and regularly engage with the culture.

### Norm-Referenced Testing Demands Standardization with Immigrant Populations

A fundamental feature underlying the creation and application of powerful psychometric tests is the standardization of results within specific populations. Indeed, large datasets provide comparators with which to assess the scores of patients relative to the population. However, a patient must belong to the population on which the standardization procedure was performed. If a patient's scores are compared to a population that is sufficiently unlike them, interpretations become effectively meaningless. Molock et al. (2023) recommend taking steps to mitigate harm caused by psychological assessments on minority groups who have historically been excluded during the creation of these tools. By doing so, culturally sensitive SRAs can be developed in ways that would be most beneficial for newcomers across multiple backgrounds.

### Culturally Specific Tools Should Be Added to Existing SRA Protocols

As suicidality is viewed, experienced, and expressed differently as a function of cultural background, the clinician's toolset should reflect this inherent heterogeneity.

One example of an existing SRA tool that addresses the needs of minority groups by incorporating cultural factors is the cultural assessment of risk for suicide (CARS) by Chu et al. (2013). The CARS was developed on the basis of their Cultural Theory and Model of Suicide (Chu et al., 2010), operationalizing and testing key factors outlined previously: cultural sanctions, idioms of distress, minority stress, and social discord. CARS displayed high internal consistency and was externally validated with items from the Beck Depression Inventory and related tests. It was also demonstrated to be an effective tool for both minority and non-minority populations, providing additional information predictive of suicide in all cases.

Beyond the development of psychometric tests, we recommend that clinicians should practise empathetic cultural awareness when listening to their patients verbally report their mental states. In addition to listening for classic warning signs and risk factors of suicide (Rudd et al., 2006), culturally specific idioms of stress should be noted. Indeed, clinical researchers could facilitate the identification of culturally specific idioms and the use of emotionally valent language by compiling clinical datasets of self-reports and examining how well they predict self-harm and suicide. In principle, the verbal content of interviews could then be compared against national and even international datasets to assess suicide risk on the basis of language alone (Shin et al., 2022).

During interviews, clinicians should also consider protective factors that are most relevant to the patient's cultural background such as connections to family and community, identity, spiritual connections, sense of belonging, and access to firearms or other highly lethal means of suicide (Sánchez, 2001). We recommend that clinicians consider the balance between being culturally sensitive and “othering” their patients. Patients should not be considered *only* by their ethnoracial and/or cultural identities but also as individuals with unique needs and risk profiles. However, considering cultural factors in suicide assessment may be a matter of life or death, and therefore the risk of othering should be balanced against the benefit of reducing suicidal behaviour. Clinicians must consider multiple dimensions of analysis simultaneously, evaluating the relevance of culture at every stage of their assessment. Indeed, because culture is embedded within language, all reports of subjective experiences are likely to be touched by a patient's unique, lived experience.

## Conclusion

Suicide and self-harm can be predicted and mitigated; however, in multicultural societies, special cultural considerations are necessary if the goal is to ensure equal access to potentially life-saving care. The power of a psychometric tool, or any assessment strategy, can be undercut by its failure to incorporate elements of culture which, as we have discussed, are highly relevant. While there has been a push for cultural consideration in psychosocial interventions (Goldston et al., 2008) and general suicide prevention models (Bowden et al., 2020), there is still a need for these considerations to be further incorporated into existing, or novel, SRA tools. Fortunately, we are seeing several tools that have been designed to accommodate the needs of immigrant populations from minority cultures, focusing on cultural sanctions, idioms of distress, minority stress, and social discord. As personalized medicine becomes increasingly popular, and in consideration of the specificity of cultural contexts, it may be necessary to design and assemble tests and other tools to address the particular subcultures of the region within which mental healthcare is being provided.
